# Recent Advances in Research on the Effect of Physicochemical Properties on the Cytotoxicity of Metal–Organic Frameworks

**DOI:** 10.1002/smsc.202200044

**Published:** 2022-08-16

**Authors:** Fang Hao, Zhu‐Ying Yan, Xiu‐Ping Yan

**Affiliations:** ^1^ State Key Laboratory of Food Science and Technology Jiangnan University Wuxi 214122 China; ^2^ International Joint Laboratory on Food Safety School of Food Science and Technology Jiangnan University Wuxi 214122 China; ^3^ Key Laboratory of Synthetic and Biological Colloids Ministry of Education Jiangnan University Wuxi 214122 China

**Keywords:** cellular toxicity, exposure routes of MOFs, metal–organic frameworks, physical and chemical characteristics of MOFs

## Abstract

Metal–organic frameworks (MOFs) have been utilized with increasing interest in various fields, including gas storage and separation, catalysis, sensing, adsorption, and biomedicine. Recently, the scale‐up production and commercialization of MOFs have paved their way to real‐world applications. However, the accidental and intentional exposures of MOFs to humans and organisms make an increasing concern on their health risks and sustainable development. Thus, toxicity assessment is essential for the application of MOFs. In vitro toxic evaluation based on cell culture is low cost, fast, and high throughput, making it an ideal model in toxicity research. To understand the cytotoxicity of MOFs, a short review on the effect of key physicochemical factors on cytotoxicity is necessary. Herein, the application of MOFs is summarized and the possible exposure routes of MOFs to humans are discussed. Moreover, the key physicochemical factors affecting the cytotoxicity of MOFs such as chemical composition, size, and shape are also elucidated. It is expected that this short review helps to understand the cytotoxicity of MOFs and sheds light on the importance of the toxicity assessment of MOFs.

## Graphical abstract

The wide application of metal–organic frameworks (MOFs) has led to environmental health risks to humans. Understanding the toxicity of MOFs is essential for its sustainable development. Herein, the representative applications of MOFs are summarized and the potential environmental exposure routes of MOFs to humans are proposed. The key physicochemical factors of MOFs affecting cytotoxicity are discussed in detail.

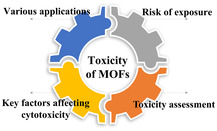

## 1 Introduction

Reticular chemistry has become a powerful tool for scientists to design crystalline porous materials with different fundamental units.^[^ ^1^ ^]^ Over the past decades, porous materials have gained great attention due to their outstanding performance and wide applications. Among various porous materials, the combination of metal nodes and organic linkers by coordination bonds creates new hybrid porous materials called metal–organic frameworks (MOFs) and opens a new window in the design of porous materials.

Since the first report on MOFs by Yaghi's group in 1995,^[^ ^2^ ^]^ more than 100 000 kinds of MOFs have been synthesized according to the Cambridge Structural Database (CSD). The unique characteristics, including tunable porosity, customized chemical composition, large specific surface area, and easy functionalization, make MOFs promising candidates for numerous applications in diverse fields such as gas storage and separation, catalysis, sensing, adsorption, energy, and biomedical sciences.^[^ ^3^ ^]^ In addition, the large‐scale production and commercialization of MOFs began from 2016.^[^ ^4^ ^]^ For instance, the commercial MOF adsorbent for storage of 1‐methylcyclopropene to ripen vegetables and fruits was driven to the market.^[^ ^5^ ^]^ The Numat's ION‐X gas cylinder was designed based on the MOF adsorbent to store hazardous gases for semiconductor industry, showing significant advantages over traditional adsorbents.^[3a]^ Apart from the commercialization in gas storage, the MOF‐303‐based apparatus has been used to collect water in the desert.^[^ ^6^ ^]^

Despite the advantages of MOFs, the wide application of MOFs has led to environmental health risks. The accidental release of MOF particles into the environment from their bulk‐products is inevitable during the lifecycle of MOFs. The deposition, decomposition, and distribution of released MOFs in environmental media could result in the exposure to humans and other organisms, inducing various biological effects. In addition, the intentional administration of MOFs in biomedicine (such as bioimaging, drug delivery system) also leads to the exposure risks to humans. Under these circumstances, the environmental health risks of MOFs are unavoidable.

The toxicity of MOFs plays an important role in their health risk assessment. Compared with in vivo and ex vivo toxicity assessments, in vitro toxicity evaluation based on cell culture is low cost, fast, and high throughput, making it an ideal model in toxicity research. As the basic unit in organism, cells play an important role in life activity. The toxic effects on cells would affect their function and therefore induce significant response to humans. Thus, cytotoxicity evaluation is the basic step in health risk assessment.

Several excellent reviews on synthesis, design, and application of MOFs have been published. For example, Stock's group provided a summary about the synthesis routes of MOFs with various topologies, morphologies, and composites.^[^ ^7^ ^]^ Zhou and co‐workers summarized the recent development in design and synthesis of multicomponent MOFs.^[^ ^8^ ^]^ Besides, a few reviews concentrated on biomedical application of MOFs, discussed the importance in exploring the toxicity of MOFs, and clarified the guideline for nanosafety evaluation of MOF nanoparticles. For instance, Horcajada's group summarized the biorelated applications of MOF nanoparticles and thought the toxicity of MOFs should be considered before applications.^[^ ^9^ ^]^ Wuttke's group showed the state of the‐art in nanosafety evaluation of MOF nanoparticles and discussed the relevance of toxicity in the context of application.^[^ ^10^ ^]^ However, the reviews on the risk of environmental exposure of MOFs and the effect of physicochemical properties on the cytotoxicity of MOFs are still hard to find.

This review aims to clarify the environmental exposure routes of MOFs, highlight the recent progress on the cytotoxicity of MOFs, and elucidate the key physicochemical factors affecting the cytotoxicity of MOFs. The perspective is also proposed for overcoming the challenges in assessing cytotoxicity of MOFs. We expect that this review will help to understand the toxicity of MOFs and provide an inspiration for sustainable development of MOF industries.

## 2 Universal Applications for MOFs

**Figure 1:**
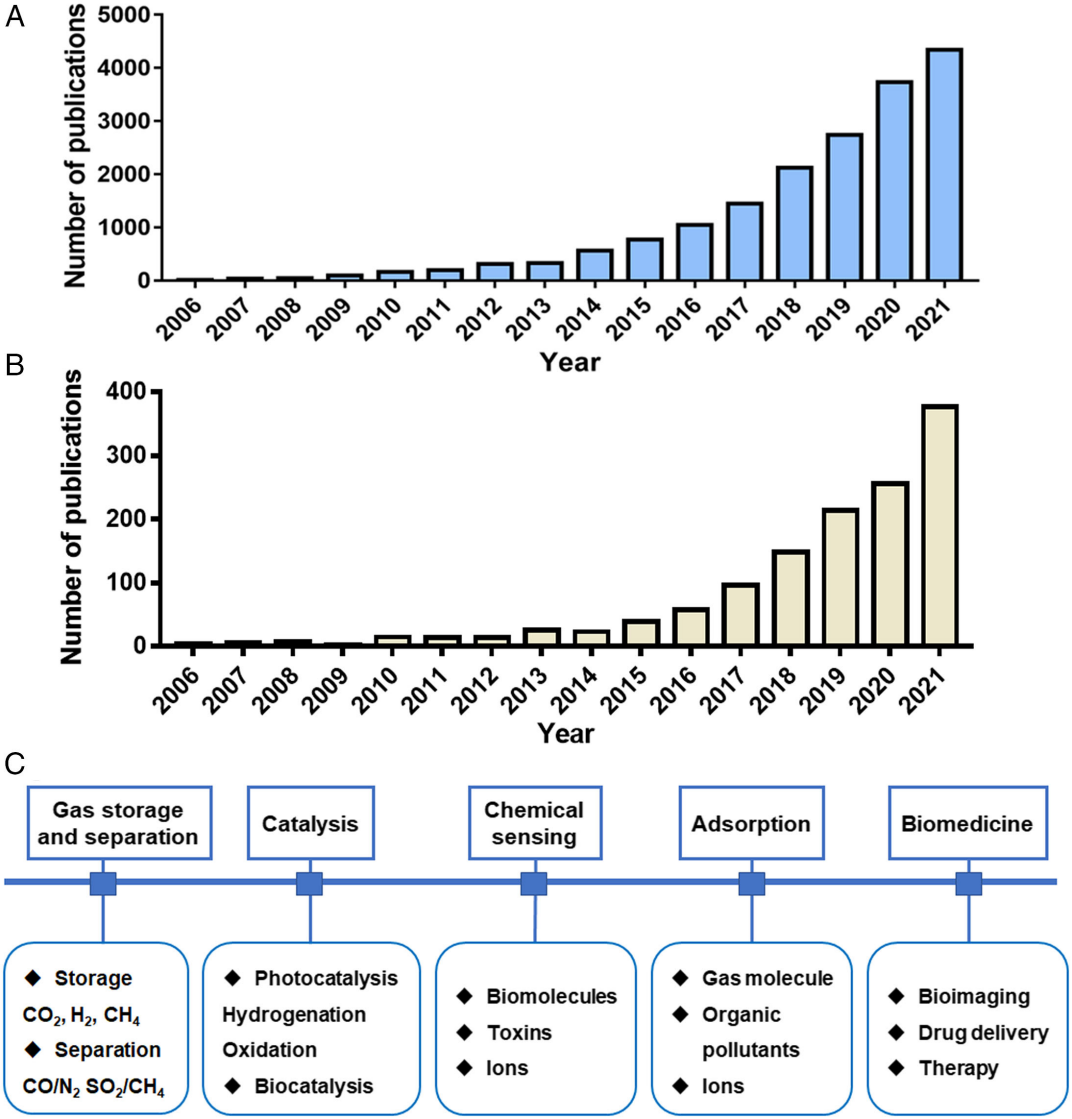
Growth trend of published articles on A) the applications of MOFs and B) toxicity of MOFs from 2006 to 2021 (data from Web of Science). C) Various applications of MOFs.

The number of research publications related to the application of MOFs (**Figure** **1** **A**) as well as the study on the toxicity of MOFs (Figure 1B) has been increasing continuously. Extensive studies have been performed to obtain functional MOFs for further applications. The prepared MOFs have been mainly used in gas storage and separation, catalysis, sensing, adsorption, and biomedicine (Figure 1C). In this section, we will highlight the representative studies in this field to emphasize the necessity for the safety assessment of MOFs as the wide utilization of MOFs will increase the environmental health risks.

### 2.1 Gas Storage and Separation

MOFs have advantages in gas storage and separation due to their high porosity and open metal sites (OMS). Tuning the pore topology, organic linkers, and OMS can provide an abundant number of adsorption sites and specific binding affinity to achieve high‐performance gas adsorption and separation.

A series of MOFs showed high capacity for the uptake of CO_2_, H_2,_ and CH_4_. MOF‐177 had a high capacity of 33.5 mmol g^−1^ for CO_2_ uptake at 35 bar.^[^ ^11^ ^]^ The sodalite‐like Mn‐based MOF, Mn_3_[(Mn_4_Cl)_3_(BTT)_8_(CH_3_OH)_10_]_2_, was found to yield 6.9 wt% H_2_ uptake at 90 bar.^[^ ^12^ ^]^ Besides, Ni‐based MOF‐74 had high volumetric CH_4_ uptake with 230 cm^3^ cm^−3^ at 35 bar.^[^ ^13^ ^]^ The uptake capacity of gas could be tuned by replacing OMS and changing pore topology. Exposing more OMS or high polarizing ions leads to strong electrostatic interaction between OMS and gas molecules, resulting in high capacity of gas uptake.^[^ ^14^ ^]^ Decreasing pore dimensions and increasing surface area of MOFs is also in favor of gas uptake due to the strong van der Waals interaction between gas molecules and framework.^[^ ^15^ ^]^ Another strategy to increase gas uptake is to use flexible MOFs. The channel transformation from “collapsed” to “expanded” state makes MOF breathe under the pressure change of gas, providing a steep isotherm in gas release.^[^ ^4^ ^]^

The gas separation depends on the selective adsorption of MOFs to different gases. Fe_2_Cl_2_(bbta) (bbta=1 H,5Hbenzo(1,2‐d:4,5‐d′)bistriazole) enabled separation of polarizable CO from the mixtures of CO/N_2_ and CO/H_2_.^[^ ^16^ ^]^ Al‐based NOTT‐300 was an effective adsorbent for the separation of SO_2_ from the mixture of CH_4_, N_2_, and H_2_ as the strong hydrogen bonds between SO_2_ and hydroxyl groups of NOTT‐300.^[^ ^17^ ^]^ Zr‐based UiO‐66 could selectively capture NH_3_ from air due to the strong interaction of ‐COOH and NH_3_ with adsorption capacity of 55 mg g^−1^.^[^ ^18^ ^]^

### 2.2 Catalysis

The virtues of MOFs, including high porosity, large surface area, and customized pore architecture, give them great advantages in heterogenous catalysis. There are two main applications for MOFs in catalysis. One is the utilization of their metal nodes or organic linkers as catalytic sites. The other is the encapsulating of catalysts (such as nanoparticles and enzyme) in the pore of MOFs as composite catalysts.

MIL‐100 containing Fe and Ni metal nodes exhibited a significant improvement in catalytic activity for Prins reaction in the case of the catalyst containing 5% Ni.^[^ ^19^ ^]^ Ru nanoparticles were loaded into MOF‐5 by reductive reaction. The obtained Ru@MOF‐5 catalyst was used in benzene hydrogenation and benzyl alcohol oxidation.^[^ ^20^ ^]^ A similar method was used in the synthesis of Cr‐based MIL‐101 in the hydrogenation reaction.^[^ ^21^ ^]^ The cytochrome was encapsulated in zeolite‐like ZIF‐8 to obtain the composite.^[^ ^22^ ^]^ The enzymatic activity of cytochrome in the MOF increased by ten times compared with its native counterpart. A Ti‐substituted Keggin‐type polyoxometalate [PTi_2_W_10_O_40_]^7−^ was decorated on the surface of HKUST‐1 for photocatalytic reduction of CO_2_ to CO.^[^ ^23^ ^]^

### 2.3 Chemical Sensing

The pores of MOFs could result in the preconcentration of analyst and significantly increase sensing performance. In addition, postsynthesis modification of MOFs can introduce new functionalities to MOFs to improve the selectivity. Therefore, MOFs have been widely used in sensing biomolecules, environmental toxins, ionic species, and many others.

In 2018, Schaate's group developed a Zr‐based MOF thin film for selective detection of NO_2_. The organic linker, calixarenes, was used for visual detection and encapsulation of NO_2_ through the interaction of charge transfer complexes in the MOF.^[^ ^24^ ^]^ Huang's group used a Cu‐based MOF to construct a flexible sensor to detect biomolecules such as glycine, tryptophan, and glucose with continuous monitoring capacity for 20 days.^[^ ^25^ ^]^ A kind of turn‐on fluorescent lanthanide MOF was designed by Huang’ group for sensitive and selective detection of mercury ions.^[^ ^26^ ^]^ The fluorescence of MOF was quenched by adsorption of imidazole‐4,5‐dicarboxylic acid (IDA), whereas mercury ions made strong coordinate bonds with IDA, resulting in the recovery of fluorescence. Zhou and co‐workers reported two kinds of Zr‐based MOFs (BUT‐12 and BUT‐13) to selectively detect nitrofurazone (NFZ) and nitrofurantoin (NTZ) in aqueous solution.^[^ ^27^ ^]^ The adsorption of NFZ and NTZ significantly quenched the fluorescence of BUT‐12 and BUT‐13 due to the energy transfer, giving a detection limit on the ppb scale.

### 2.4 Adsorption

Finding proper adsorbents is the core for adsorption. In recent years, researchers have confirmed that OMS on the metal nodes, functional groups on organic linkers, and modification of pores on MOFs led to enhanced selectivity and adsorption capacities for targeted contaminants due to the electrostatic interaction, hydrophobic interaction, π−π interaction, and hydrogen bonding or a combination of these interactions.^[^ ^28^ ^]^ Numerous kinds of MOFs with high adsorption capacity were synthesized for gas adsorption, organic pollutants adsorption, and ions adsorption.

In 2018, Yang and co‐workers designed Al‐based MEF‐3000 for ammonia adsorption. The as‐prepared MOFs exhibited reversible ammonia adsorption performance with the uptake of 15.7 mmol g^−1^ at 1 bar. In particular, 50 cycles of adsorption–desorption led to no loss in adsorption capacity for ammonia.^[^ ^29^ ^]^ Jhung's group used the highly stable Cr‐based MIL‐53 for the adsorption of 2,4‐dichlorophenoxyacetic acid (2,4‐D) from water. They demonstrated that MIL‐53 had a much higher adsorption capacity (556 mg g^−1^) for 2,4‐D than activated carbon (286 mg g^−1^) in just 1 h.^[^ ^30^ ^]^ Rana and co‐workers designed electrospun nanofibers containing Zr‐based MOF‐808 to adsorb cadmium and zinc ions.^[^ ^31^ ^]^ They found that the composite nanofiber had higher adsorption capacity compared with pristine MOFs, with maximum adsorption capacities of 225.05 and 287.06 mg g^−1^ for Cd^2+^ and Zn^2+^, respectively.

### 2.5 Biomedical Applications

Increasing attention has been paid to the application of MOFs in biomedical science as the confined space of reticular frameworks provides various opportunities for the applications of bioimaging, biosensing, drug delivery and therapy, and antibacterial.

#### 2.5.1 Bioimaging, Biosensing, Drug Delivery, and Cancer Therapy

MOFs have been successfully used in biomedical science, including bioimaging, biosensing, drug delivery, and cancer therapy, as their properties of flexibility, porosity, physicochemical characteristics (size, shape, surface functionalization) could be precisely designed.

The chemical‐stable ^89^Zr‐based UiO‐66 was synthesized by Hong's group for positron emission tomography (PET) imaging.^[^ ^32^ ^]^ The radioactive UiO‐66 nanoparticles were functionalized with PEG as well as the tumor‐targeting peptide ligand F3. After intravenous injection to mice, PET imaging showed fast accumulation of MOF nanoparticles in subcutaneous MDA‐MB‐231 breast cancer tumors. Similarly, the porphyrinic MOF conjugated with tumor‐targeting aptamer was synthesized for fluorescent imaging by Tan's group.^[^ ^33^ ^]^ The fluorescence of aptamer was quenched by porphyrinic MOF, but recovered after the binding of the aptamer with PTK7 protein of Hela cells, resulting in turn‐on bioimaging for specific cancer cells. Owing to the tunable luminescent properties of metal nodes and organic linkers, the MOF‐based luminescent biosensing has been explosively developed. Yang's group developed several MOF‐based fluorescent nanosensors to detect metal ions (Fe^3+^ and Cu^2+^),^[^ ^34^ ^]^ phosphate,^[^ ^35^ ^]^ and peptide^[^ ^36^ ^]^ in cells with low limit of detection. Tang's group constructed MOF‐based sensor platforms to detect intracellular molecules including phosphate and mRNA.^[^ ^37^ ^]^ Inspired by biomineralization, Chu and co‐workers encapsulated biomolecules into ZIF‐8 under mild synthesis conditions.^[^ ^38^ ^]^ The as‐prepared drug delivery platform could be internalized by cells and protect cargoes from lysis, maintaining the activity of biomolecules for therapy. The similar method was also used by Wu's group to encapsulate lysozyme for cancer therapy.^[^ ^39^ ^]^ Qu and co‐workers synthesized ZIF‐67 loaded with 3‐amino‐1,2,4‐triazole and functionalized with PEG.^[^ ^40^ ^]^ The nanodrug possessed superoxide dismutase‐mimicking activity with promotion of H_2_O_2_ and was then used as chemodynamic agent for cancer therapy. They also decorated the ZIF‐8 nanoparticles with erythrocyte membrane as biomimetic nanoreactor for colon therapy or with Cu^2+^ and DNAzyme as nanodrug for gene therapy.^[^ ^41^ ^]^

#### 2.5.2 Antibacterial Application

MOFs have emerged as promising materials for antibacterial application due to their high loading and sustained releasing capacities of antimicrobial agents, high yield of reactive oxygen species (ROS) under light irradiation, strong interaction with the membrane of bacterial, and controlled/stimulated decomposition at the infection area.^[^ ^42^ ^]^

Zn‐based ZIF‐8 is a promising candidate in antibacterial application. Wang and co‐workers fabricated ZIF‐8‐based air filter in air cleaning and found that this fibrous filter showed >99.99% efficiency of photocatalytic killing against airborne bacteria in 30 min light irradiation.^[^ ^43^ ^]^ Yang and co‐workers revealed that Cu‐based MOF‐199 made significant inactivation of Gram‐negative bacterium (*Escherichia coli*) and Gram‐positive bacterium (*Staphylococcus aureus*) at high concentration (>1000 mg mL^−1^).^[^ ^44^ ^]^ The synergistic treatment with MOF and antibiotics has been proved an efficient approach in antibacterial. Wang's group reported the synergistic system composed of ZIF‐8 and tetracycline for the elimination of intracellular bacteria.^[^ ^45^ ^]^ The released tetracycline and Zn^2+^ from the obtained system in the acidic infection area gave a synergistic antibacterial efficiency of over 98%. Qu's group developed a hybrid nanocatalyst consisting of 2D Cu‐based MOF and glucose oxidase.^[^ ^46^ ^]^ The nanocatalyst gave effectively generation of hydroxyl radial and antibacterial capacity both in vivo and in vitro.

## 3 Possible Exposure Routes of MOFs to Human

The increasing applications of MOF‐based technology and products inevitably enhance the exposure risks of humans to MOFs. The intentional exposure of MOFs to humans in biomedicine, including inhalation uptake, oral administration, transdermal treatment, and intravenous injection, results in potential exposure risks. The accidental exposure of MOFs released from bulk products may occur through inhalation, digestion, and transdermal penetration. In this section, we will discuss the possible exposure routes of MOFs to humans.

### 3.1 Inhalation

MOFs could easily become suspending particulate matter (diameter <2.5 μm) with the help of physical forces (such as shear force and extruding force), chemical reaction (decomposition and recrystallization), and biological reaction (biomineralization and metabolism). Thus, respiratory uptake is a common exposure route for MOF intake. The respiratory system is composed of airways, lungs, and muscles.^[^ ^47^ ^]^ The translung uptake is the main route for nanoparticle respiratory uptake.^[^ ^48^ ^]^ Once MOFs flow into the respiratory system, they will pass through trachea, beginning at the larynx and ending to the thorax, and then reach the bronchi, containing cartilage and mucous‐secreting glands in their walls.^[^ ^49^ ^]^ Soon thereafter, MOFs will arrive at the bronchioles, which are small airways with diameter less than 2 mm. The bronchi and bronchioles make MOFs in air stream suffer from sharp turns due to their various sizes and angulations. Finally, MOFs will reach to the interior of alveoli, the basic unit of lung formed by bronchiole narrowing.^[^ ^50^ ^]^ The alveoli are mainly composed of type I and type II alveolar epithelial cells. Type I cells adhere tightly to each other with their basement membrane to form the air–blood barrier, while type II alveolar epithelial cells secrete phospholipids to form a single‐molecular thick layer (i.e., pulmonary surfactant) coated on the alveolar surface.^[^ ^51^ ^]^

**Figure 2:**
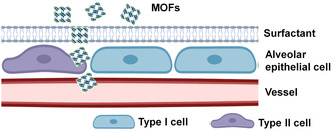
Schematic of the structure of alveoli and its uptake route of MOFs.

Considering the physiological structure of the respiratory system, the entrance of inhaled MOFs through the lung to the internal environment is that the MOFs pass through surfactant film and overcome the epithelial cell junction to reach the circulation system after escaping from the uptake of alveoli macrophages (**Figure** **2**). Previous studies showed that hydrophilic nanoparticles could quickly translocate across the surfactant film.^[^ ^52^ ^]^ Meanwhile, 20 nm TiO_2_ and 10 nm gold nanoparticles could induce significant decomposition of the epithelial junction, resulting in the formation of cellular gaps for nanoparticle transportation.^[^ ^53^ ^]^ Thus, the hydrophilic MOFs with proper sizes may have the ability to translocate the surfactant film and air–blood barrier, reaching the circulation system.

### 3.2 Digestion

The emerging MOF‐containing products, such as antibacterial agents, nanopesticides, and food additives, are capable of entering the internal environment through oral uptake. MOF‐based drug delivery system could be delivered to target organs through oral administration due to its convenience and patient compliance.^[^ ^54^ ^]^ Besides, the environmental pollutants containing MOFs are likely to enter the digestive system through drinking water. Thereafter, the digestive system is the potential exposure route for MOF intake.

**Figure 3:**
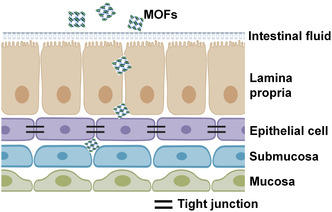
Schematic of gastrointestinal tract and its uptake route of MOFs.

The digestive system is composed of digestive tract and glands. The tract consists of the oral cavity, the pharynx, the esophagus, and the gastrointestinal tract. Meanwhile, glands include salivary glands, the liver, and the pancreas. The gastrointestinal tract is composed of four layers: the innermost layer, mucosa; the connective layer, submucosa that supports mucosa; the epithelial cell layer surrounding submucosa; and the outmost layer, lamina propria.^[^ ^55^ ^]^ Among the four layers, the mucosa with secreted gastrointestinal fluid and the tight junction constructed by epithelial cells are the two main barriers for the access of MOF to the circulation system. Therefore, the internal exposure route for MOF is to cross these barriers, including translocating across mucosa and overcoming the tight junction (**Figure** **3**).

Several MOF‐based oral drug systems have been demonstrated to overcome gastrointestinal barrier. Horcajada's group reported that Fe‐based MIL‐127 with a diameter of 300 nm could penetrate the gastrointestinal barrier to reach the circulation system after oral uptake while the integrity of stomach and intestine was not affected,^[^ ^56^ ^]^ suggesting the trans‐gastrointestinal pathway for the translocation of MIL‐127. They then found that Fe‐based MIL‐100 had high loading efficiency of genistein and great bioavailability from gastrointestinal tract to blood.^[^ ^57^ ^]^ The potential for oral uptake of MIL‐100 was also investigated by Chen's group, who used MIL‐100 to encapsulate insulin for diabetic therapy.^[^ ^58^ ^]^ They found that MIL‐100 could enter into blood and increase the retention time of insulin. Another research further confirms that the surface functionalization of biopolymer chitosan on 300 nm MIL‐127 significantly increased the intestinal penetration efficiency.^[^ ^59^ ^]^ Therefore, the MOFs with biocompatible surface modification may reach the internal environment through the digestive system.

### 3.3 Skin Penetration

Various kinds of MOF‐based consuming and biomedical products have been invented. For instance, MOF‐based antibacterial agents were used in lesion location to promote skin wound healing. MOF‐based films were utilized in the package of food to extend the expiration time. The usage of MOF‐based clothing, package, skin products, and transdermal drug delivery system will lead to the exposure of skin to MOFs.

Skin is the largest organ in humans, maintaining normal physical conditions and defending environmental pollutants. Skin is composed of epidermis, dermis, and affiliated follicle/gland. Epidermis is the outmost layer of skin and contains four types of sublayers (stratum corneum, stratum granulosum, spinous layer, and basal layer from top to the bottom). Each sublayer is derived from the differentiation of keratinocytes with the surrounding of lipid and extracellular protein, providing substantial protection against exogenous substances. Thus, epidermis is the main barrier for the transdermal penetration of MOFs.

**Figure 4:**
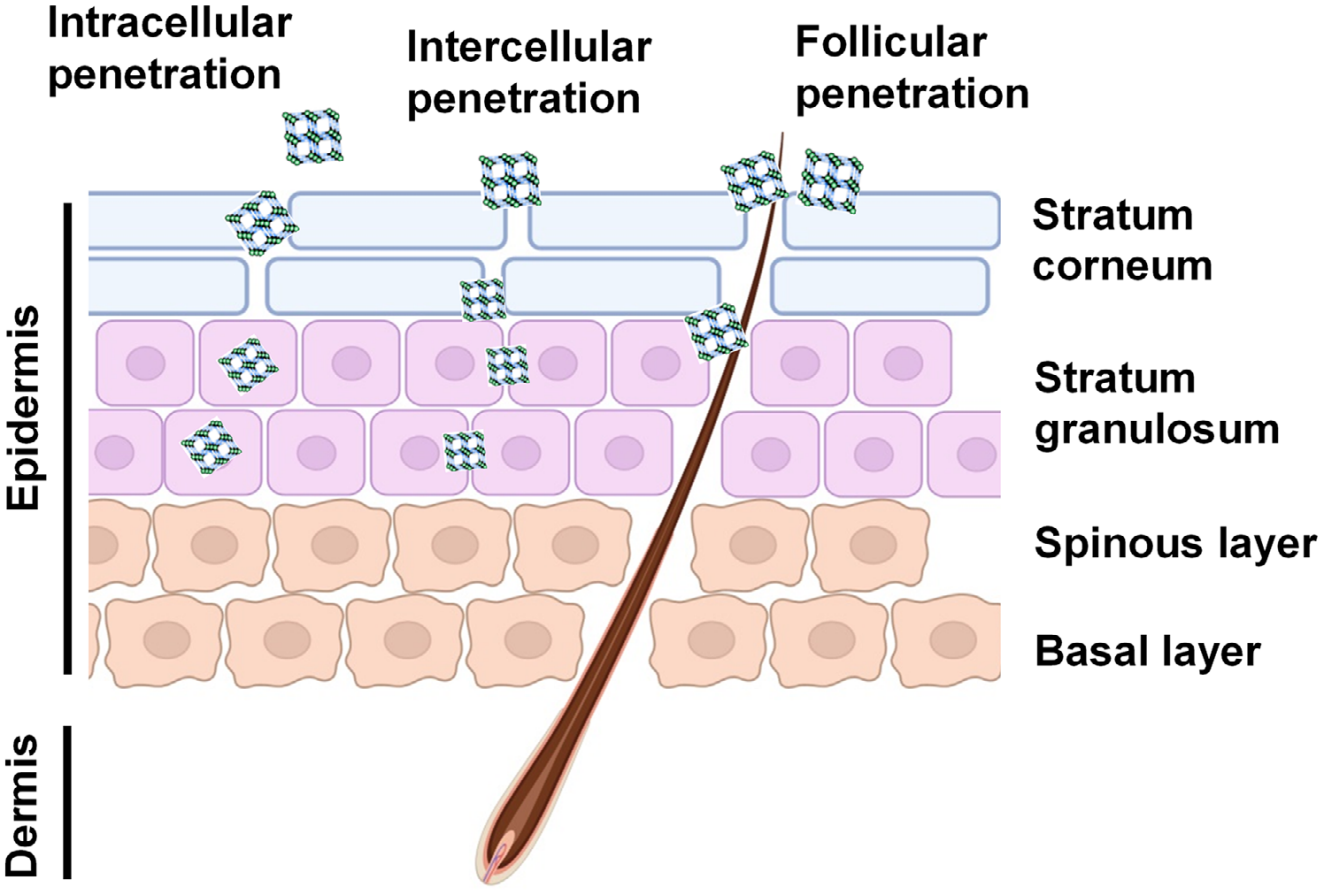
Schematic of the structure of skin and its uptake route for MOFs.

Three potential pathways for the transdermal penetration of nanoparticles have been revealed: intracellular penetration (nanoparticles utilize endocytosis and exocytosis to translocate across epidermis), intercellular penetration (nanoparticles bypass epidermal cells through cellular gaps), and follicular penetration (nanoparticles penetrate into deep dermis along the margin of hair follicle). Thus, MOFs may penetrate into skin through these pathways (**Figure** **4**).

The transdermal penetration across the epidermis of nanoparticles has been reported. Au nanoparticles penetrated across epidermis through intercellular pathway,^[^ ^60^ ^]^ while SiO_2_ could penetrate into the epidermis through follicular pathway.^[^ ^61^ ^]^ In addition, Sc‐based MOF, MFM‐300, could penetrate across epidermis and reach dermis.^[^ ^62^ ^]^ Considering that the positively charged nanoparticles have higher penetration efficiency compared with negatively charged nanoparticles, the exposure of MOFs with positive charge may induce epidermal penetration, leading to internal exposure risks.

### 3.4 Intravenous Injection

The intravenous injection is the most common therapeutic method with advantages of high bioavailability, rapid accessibility, low irritation, and so on, making it a promising method for cancer therapy.^[^ ^63^ ^]^ Patterson and co‐workers synthesized RGD‐functionalized MOF to deliver heparin and doxorubicin for treatment of lung cancer.^[^ ^64^ ^]^ They found the obtained MOF significantly decreased the volume of lung tumor in mice after intravenous injection. Meng and co‐workers synthesized cysteine‐decorated Zr‐based MOF to load cisplatin for lung tumor therapy and the nanodrug could significantly improve the therapeutic effect of cisplatin‐resistant lung cancer in mice.^[^ ^65^ ^]^ Despite the preclinical animal studies, the first clinical anticancer drug, RiMO‐301 (Hf‐based MOF with high ability of X‐ray absorption), was initiated in the phase I clinical trial for human tumor treatment with intratumoral injection.^[^ ^66^ ^]^ The other kinds of MOF nanodrugs, like CPI‐100 and CPI‐300, were also in clinical or preclinical trials as revealed by the ClinicalTrials website (https://clinicaltrials.gov/ct2/home). The clinical application of MOFs brings up the direct exposure risk to humans after intravenous injection. Besides, the injected MOFs will circulate with blood circulation and distribute to organs such as lungs, liver, inducing potential toxic concerns on molecular, cellular, and organic levels.

## 4 Key Factors Affecting the Cytotoxicity of MOFs

The physicochemical property of MOFs is emphasized while the toxic property is often neglected.^[^ ^10^ ^]^ The assessment of the toxic effect of MOFs is helpful to avoid the hazardous effect of MOFs on ecosystem and organism and improve the risk management of MOFs in different applications. Meanwhile, understanding the toxic property of MOFs could foster the “safe‐and‐sustainable‐by‐design” approach in their industrial‐production.

Over the past decades, in vitro and in vivo studies with various kinds of MOFs were performed. The in vitro model can facilitate a high‐throughput analysis of cytotoxicity without ethic issues. Besides, cytotoxicity evaluation is low cost and rapid, making it an important method for toxicity assessment.

**Figure 5:**
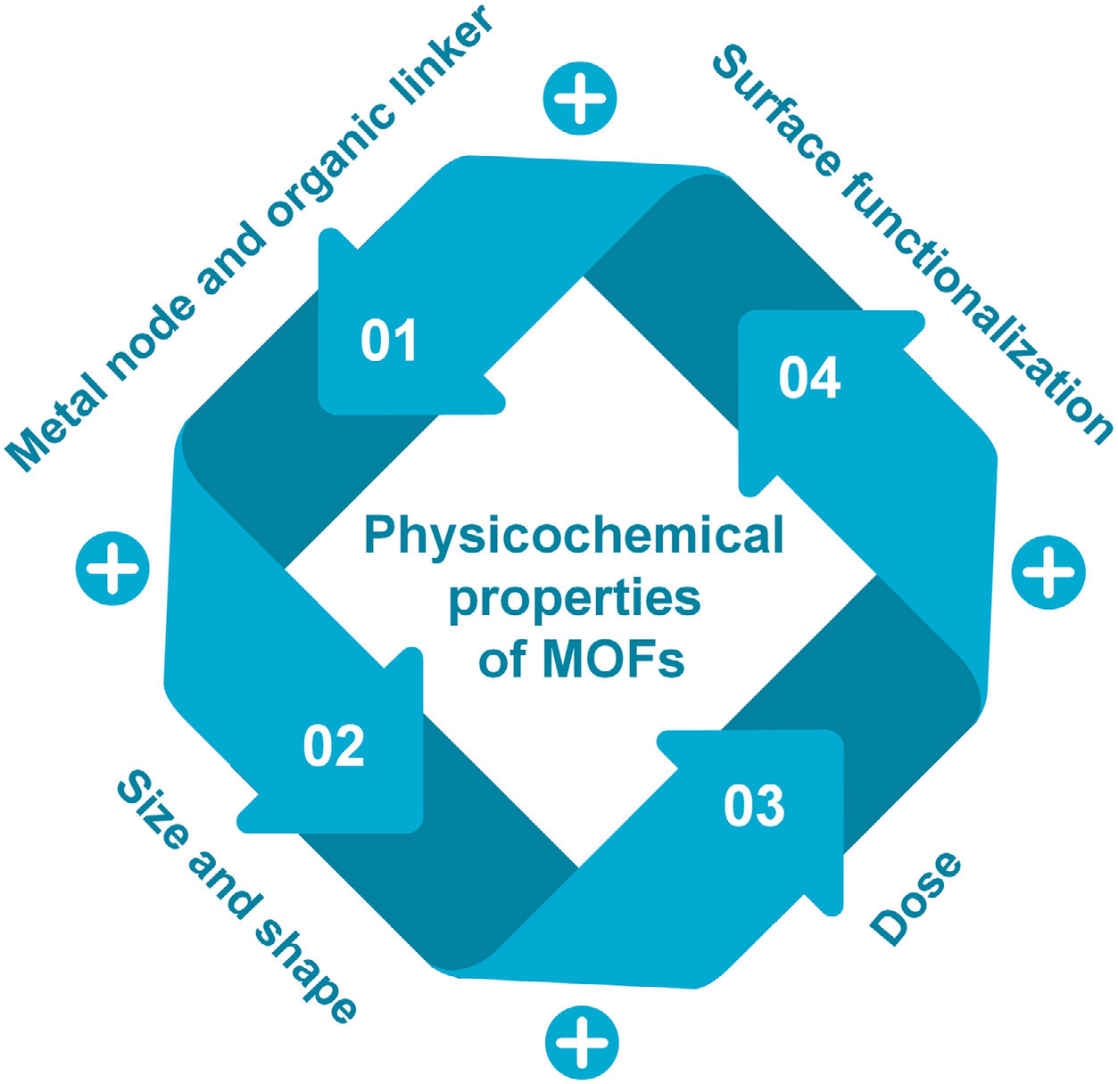
Key physicochemical factors affecting the cytotoxicity of MOFs.

The cytotoxic effect of MOFs is highly dependent on their physicochemical property. In this section, we will discuss the key factors that affect the cytotoxicity of MOFs (**Figure** **5**) and summarize the studies on the cytotoxicity of MOFs.

### 4.1 Metal Node and Organic Linker

The important principle for the toxicity study of one material is to consider its constituent toxicity because the decomposition of the material can execute under many kinds of circumstances. As MOFs are composed of metal nude and organic linker, the cytotoxicity study could not avoid both of the constituents.

#### 4.1.1 Effect of Metal Node

It is desirable to use the MOFs with the same organic linker but different metals to evaluate the effect of metal node. Thus, the MOFs with topological structures of ZIFs (ZIF stands for zeolitic imidazolate frameworks) and MILs (MIL stands for Material Institute Lavoisier) are ideal candidates.

In 2015, Horcajada's group explored the cytotoxicity of Fe(III)‐ and Cr(III)‐based MIL‐100 with the diameter of ≈140 nm.^[^ ^67^ ^]^ Four kinds of cell lines, including lung cells of A549 and Calu‐3, and hepatic cells of HepG2 and Hep3B, were selected in view of different exposure routes. The toxic effects were measured in the aspect of cell impedance, cell viability, ROS generation, and DNA damage. The results showed that Cr(III)‐based MIL‐100 did not induce toxic effects on these cell lines, but Fe(III)‐based MIL‐100 gave significant cell death and loss of cell impedance in Hep3B cells as well as the high accumulation of ROS and DNA damage. In 2022, they further studied the toxic effects of Fe(III)‐ and Al(III)‐based MIL‐100 on J774A.1 and HL‐60 cells.^[^ ^68^ ^]^ Al(III)‐based MIL‐100 induced higher ROS than Fe(III)‐based MIL‐100 in both cells in 24 h. The same trend was also observed in complement system activation assays. In addition, only Fe(III)‐based MIL‐100 significantly elevated the cytokine secretion of TNF‐α, L‐10, and IL‐1β in HL‐60 cells, suggesting the immunologic regulation of metal nodes of MOFs.

**Figure 6:**
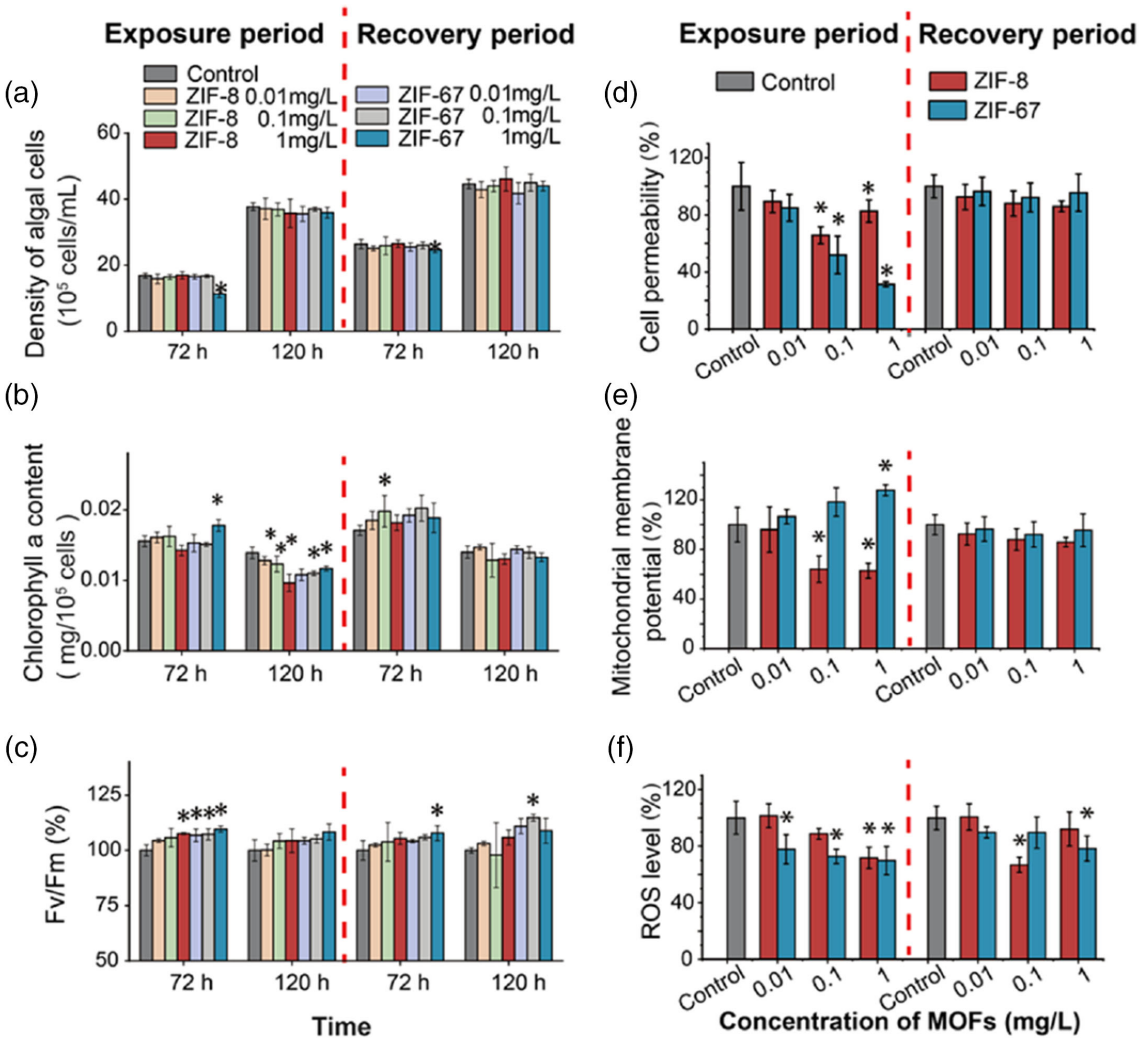
Cytotoxicity and recovery of algae exposed to ZIF‐8 and ZIF‐67. a) Density of algae; b) chlorophyll a content of the algae; c) *F*_v_/*F*_m_ value of the algae exposed to ZIF‐8 or ZIF‐67; d) cell permeability; e) mitochondrial membrane potential; and f) ROS level. Reproduced with permission.^[69a]^ Copyright 2021, American Chemical Society.

The toxic effects of Zn(II)‐based ZIF‐8 and Co(II)‐based ZIF‐67 on plant cells were studied by Hu's group.^[^ ^69^ ^]^ ZIF‐67 at low concentrations (0.01–1 mg mL^−1^) significantly inhibited the growth of algal and chlorophyll biosynthesis accompanied with the reduction of ROS while ZIF‐8 was nontoxic. The transcriptional omics studies revealed that the downregulation of oxidative phosphorylation and the adenosine triphosphate (ATP) synthesis inhibition of chlorophyll in mitochondria were responses to these toxic effects (**Figure** **6**). Meanwhile, ZIF‐67 induced a more serious metabolism perturbation than ZIF‐8 as the glycine and aspartic acid pathways were upregulated and the phenylalanine, alanine, asparagine, ornithine, and galactose pathways were downregulated.

Apart from the phytotoxicity of ZIF‐8 and ZIF‐67, Yan's group evaluated the toxic effect of ZIF‐8 and ZIF‐67 on erythrocytes.^[^ ^70^ ^]^ ZIF‐67 induced significant hemolysis and membrane rupture due to the generated ROS, whereas ZIF‐8 was hemocompatible. The toxic effects of ZIF‐67 on hemoglobin induced remarkably damage in the structure and function of hemoglobin.

Other kinds of MOFs were also studied to evaluate the effect of metal nodes on toxicity. Masphoch's group synthesized Zn(II)‐, Cu(II)‐, Ni(II)‐, Co(II)‐, Mn(II)‐, Mg(II)‐based MOF‐74 and revealed their cytotoxicity toward MCF7 and HepG2 cell lines.^[^ ^71^ ^]^ The ranking order of cytotoxicity was Mn‐based MOF‐74 > Cu‐based MOF‐74 > Zn‐based MOF‐74 > Mg‐based MOF‐74, and Ni‐based MOF‐74 > Co‐based MOF‐74. Morales's group evaluated the cytotoxicity of Zn(II)‐based CIM‐80 and Al(III)‐based CIM‐84 and found both of them did not show any toxicity.^[^ ^72^ ^]^

Collectively, the biocompatible metal (such as Mg, Ni) induces less cytotoxicity, while the transition metal (such as Co, Cu, Mn) with high ROS generation or coordination ability with biomolecules makes strong cytotoxicity. However, one cannot predict the actual cytotoxicity of MOFs only based on the metal node due to the complexity of MOF toxicity.[^1^, ^73^]

#### 4.1.2 Effect of Organic Linker

MOFs composed of various organic linkers but the same metal nodes are ideal to evaluate the effect of organic linkers on cytotoxicity. A great number of studies have been made to analyze the cytotoxicity of MILs and UiOs with different organic linkers.

The pioneer work on the effect of organic linkers on cytotoxicity was reported in 2014.^[^ ^74^ ^]^ Horcajada and co‐workers synthesized a series of Fe‐based MOFs, including MIL‐100, MIL‐101‐NH_2_, MIL‐101‐2CH_3_, MIL‐88A, MIL‐88B, MIL‐88B‐CH_3_, MIL‐88B‐NO_2_, and MIL‐88B‐2CF_3_, and tested the half maximal inhibitory concentration (IC_50_) using cell viability assays.^[^ ^74^ ^]^ They found that the hydrophobic–hydrophilic balance (log P) of the organic linker had the linear correlation with the IC_50_ of the corresponding MOFs, suggesting that the organic linker of MOFs plays an important role in the toxicity of Fe‐based MOFs.

Cui and co‐workers synthesized two kinds of Zn‐based MOFs, ZJU‐64 and ZJU‐64‐CH_3_, whose organic linkers were [1,1′:4′,1″‐terphenyl]‐4,4″‐dicarboxylic acid (H_2_TP) and 2′,5′‐dimethyl‐[1,1′:4′,1″‐terphenyl]‐4,4″‐dicarboxylic acid (H_2_TP‐CH_3_), respectively.^[^ ^75^ ^]^ Cell viability assay revealed that ZJU‐64 gave slightly higher toxicity than ZJU‐64‐CH_3_. A similar study was performed by Morales and co‐workers to study the toxicity of Zr‐based UiO‐64 and UiO‐66 (organic linkers are 2‐butenedioic acid and benzene‐1,4‐dicarboxylic acid, respectively) on J774A.1 cells.^[^ ^72^ ^]^ Both of these two MOFs had no toxic effect despite different organic linkers.

In summary, the effect of metal node and organic linker on cytotoxicity reveals that the less toxic building blocks may reduce the toxicity of bulk MOFs especially when MOFs released the metal and organic linker in cells. However, it is important to note that one can roughly predict the trend of the toxicity of MOFs but the toxic property of each MOF should be evaluated individually.

### 4.2 Effect of Size and Shape

Decrease in particle size results in a significant increase in the outer surface area, leading to a greater proportion of atoms being exposed to the biological environment. Thus, the smaller MOFs have higher chemical reactivity and in turn a faster degradation rate in cells. In addition, the size of MOFs affects the efficiency of cellular internalization. However, the relationship between size and efficiency of internalization is sophisticated as revealed by previous studies, leading to complex toxic outcomes.^[^ ^70^, ^76^ ^]^ Therefore, identifying the effect of size on cytotoxicity is important.

**Figure 7:**
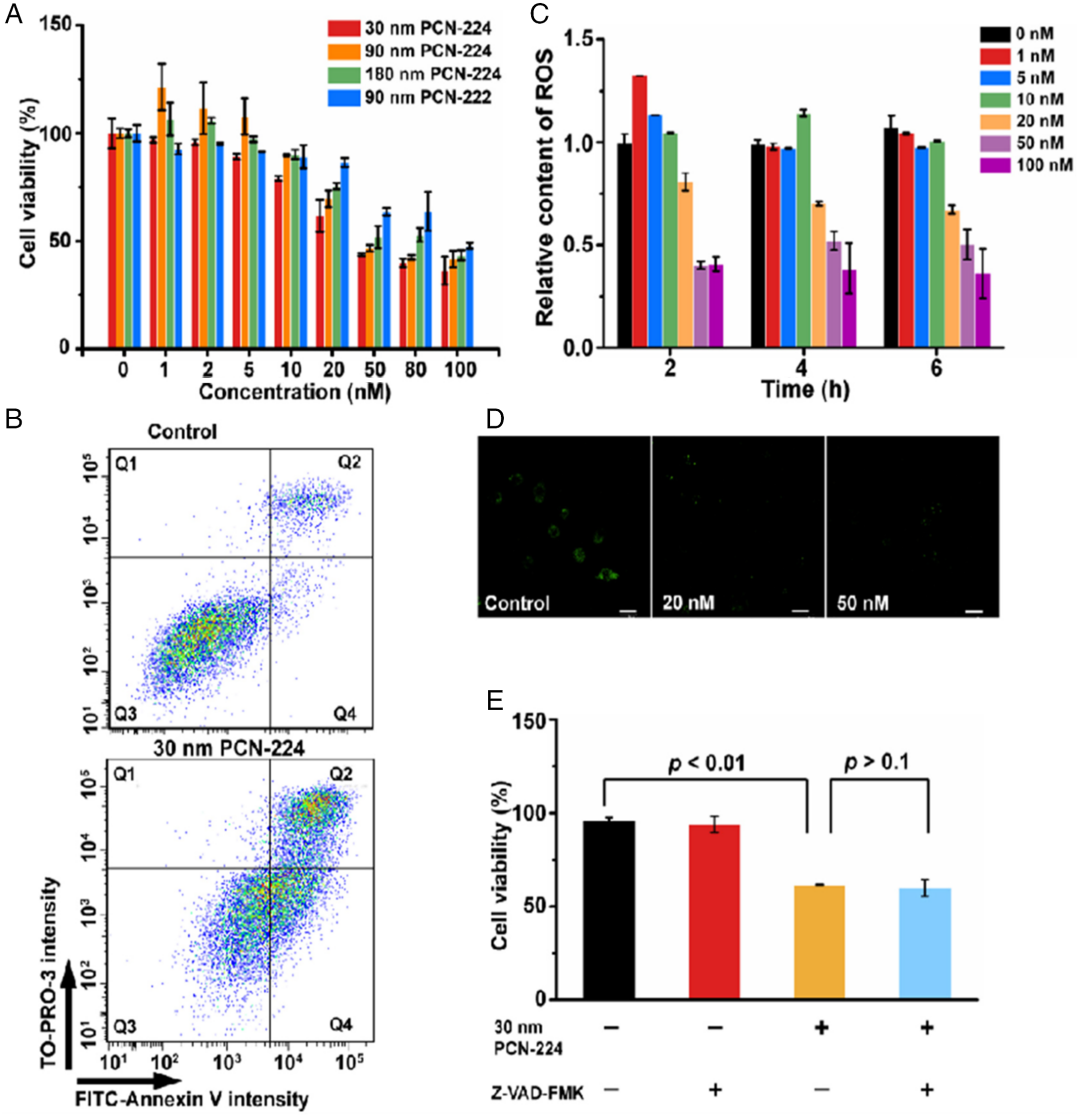
Cytotoxicity of PCN‐224 and PCN‐222. A) Viability of J774A.1 cells treated with different concentrations of PCNs for 24 h. B) Flow cytometry for cell death analysis using Annexin V‐FITC and TP3 staining. C) Relative content of ROS in J774A.1 cells treated with 30 nm PCN‐224 compared with negative control (PBS‐treated cells) at 2, 4, and 6 h. D) LSCM images of activated caspase 3/7 in J774A.1 cells after treatment with 30 nm PCN‐224 (20 and 50 nM) for 12 h. Scale bars, 20 μm. E) Viability of J774A.1 cells treated with 30 nm PCN‐224 (20 nM) for 24 h with or without caspase inhibitor Z‐VAD‐FMK (20 μM). Reproduced with permission.^[^ ^77^ ^]^ Copyright 2022, Elsevier.

Size‐controlled synthesis of Zr‐based PCN‐224 MOFs was reported by Zhou's group in 2016. The phototoxicity of 30, 60, 90, 140, and 190 nm PCN‐224 toward Hela cells exhibited a U‐shaped correlation between size and cytotoxicity, in which 90 nm PCN‐224 gave the strongest phototoxicity compared to other MOFs.^[^ ^76^ ^]^ Subsequently, Yan's group evaluated the dark toxicity of 30, 90, and 180 nm PCN‐224.^[^ ^77^ ^]^ The results showed that smaller PCN‐224 gave higher cytotoxicity due to the stronger phospholipid adsorption and higher cellular dissolution, which induced significant cell necrosis (**Figure** **7**).

Meiners's group investigate the cytotoxicity of two different sizes of Zr‐fumarate (Zr‐fum) MOF.^[^ ^78^ ^]^ The large Zr‐fum (130 nm) reduced the metabolic activity of human Schwann cell and inhibited the viability of fibroblast cells whereas the small Zr‐fum (83 nm) had no obvious toxicity. Forgan's group then used 23 nm and 168 nm Zr‐fum to evaluate their cellular internalization and cytotoxicity.^[^ ^79^ ^]^ 23 nm Zr‐fum gave higher uptake efficiency and stronger inhibition of cell proliferation compared with 168 nm Zr‐fum.

The cytotoxicity of micro‐ and nanosized Mg‐based MOF‐74 was explored by Pei's group.^[^ ^80^ ^]^ 3.7 μm MOF‐74 made high cell apoptosis with the IC_50_ of 798 μg mL^−1^, while 300 nm MOF‐74 gave the IC_50_ more than 2000 μg mL^−1^, suggesting that nanosized MOF‐74 was more biosafe than microsized MOF‐74. Further exploration for the source of toxicity then confirmed that the microsized MOF‐74 could not enter into cells, resulting in the imbalance of osmotic pressure on the physiological function of the cell membrane.

The cytotoxic effect of different sizes of ZIF‐8 on HepG2 was studied by Hu's group.^[^ ^81^ ^]^ 50 nm ZIF‐8 induced highest rate of cell necrosis compared with 90 and 190 nm ZIF‐8. The ROS generation, DNA damage, and inflammation induced by Zn^2+^ released from 50 nm ZIF‐8 were the most significant than 90 and 190 nm ZIF‐8.

The effect of shape on cytotoxicity has not been investigated in detail. One example for this effect was reported by Yan's group.^[^ ^82^ ^]^ They synthesized spherical PCN‐224 and rod‐shape PCN‐222 with the same building blocks (Zr nodes and meso‐tetra(4‐carboxyphenyl) porphyrin) and similar hydrodynamic sizes. PCN‐224 gave stronger inhibition of cell viability and higher rate of cell necrosis than PCN‐222. The photocytotoxicity of four kinds of MOFs composed of Zr and TCPP, including cubic MOF‐525, rod‐shaped MOF‐545, spindle‐shaped PCN‐223, and spherical PCN‐224, toward *Escherichia coli* (*E. coli*) and *Staphylococcus aureus* (*S. aureus*) was compared.^[^ ^83^ ^]^ An cytotoxicity order of MOF‐545 > MOF‐525 > PCN‐224 > PCN‐223 was observed, indicating the remarkable effect of shape on cytotoxicity.

Collectively, the size and shape of MOFs affect the cellular response in the aspect of cell viability, ROS generation, DNA damage, and inflammation, resulting in different cytotoxic effects. Further studies are also needed to verify the regulating patterns of size/shape on cytotoxicity.

### 4.3 Surface Functionalization

Surface properties regulate the interaction between MOFs and biological environment. External surface impedes the direct contact of biomolecules and building blocks of MOFs, in turn tunes the stability of MOFs and reduces the formation of biomolecular corona, and changes the cellular response of MOFs. As a result, the intracellular fate of surface‐functionalized MOFs as well as their cytotoxicity may be changed.

The cytotoxicity of surface‐functionalized MILs is being studied since 2015 as MILs, including Fe‐based MIL‐100 and MIL‐101, are the most commonly used MOFs in biomedical sciences. Horcajada's group confirmed that Fe‐based MIL‐100 with heparin functionalization could decrease the cytotoxicity of macrophages due to the reduction of cellular ROS generation and inflammation whereas bare MIL‐100 gave significant cytotoxicity.^[^ ^84^ ^]^ They also found that chitosan‐functionalized MIL‐100 could reduce the immunological response of human peripheral blood mononuclear cells (PBMCs) compared with bare MIL‐100 though both have negligible effect on viability.^[^ ^85^ ^]^ Besides, the cytotoxicity of lipid‐functionalized MIL‐100 and bare MIL‐100 was investigated by Wuttke's group.^[^ ^86^ ^]^ The lipid‐functionalized MIL‐100 decreased the viability of Hela cells with an IC_50_ of 3 μg mL^−1^. However, the bare MIL‐100 did not show any toxicity to cells due to its low cellular internalization. On the contrary, the 1,2‐dioleoyl‐sn‐glycero‐3‐phosphocholine (DOPC)‐functionalized MIL‐101 exhibited lower toxicity in ROS generation, membrane rupture, and cell viability than bare MIL‐101.^[^ ^78^ ^]^ Meanwhile, DOPC functionalization significantly decreased the cellular inflammatory response of MIL‐101.

Apart from the functionalized‐MILs, Zn‐based ZIF‐90 functionalized with –NH_2_, –COOH, and –SH was synthesized.^[^ ^87^ ^]^ The cytotoxicity of these ZIF‐90s was tested and ZIF‐90‐NH_2_ had the highest toxic effects, followed by ZIF‐90‐COOH and ZIF‐90‐SH, as revealed by cell viability. Zr‐based UiO‐66 functionalized with –NH_2_, –OH, and bare UiO‐66 exhibited various toxic effects on erythrocytes.^[^ ^88^ ^]^ Bare UiO‐66 induced strongest hemolysis compared to UiO‐66‐OH and UiO‐66‐NH_2_, suggesting that the surface functionalization reduced the cytotoxicity of UiO‐66. Yang's group used Cu‐based bare HKUST‐1 and carbon‐functionalized HKUST‐1 to investigate the toxicity to fungi cells.^[^ ^89^ ^]^ They found that carbonization was an efficient way to reduce the toxicity of HKUST‐1 as it reduced the damage to mycelia and peroxidase enzymes.

To sum up, the surface functionalization with negative charge could inhibit the interaction with cells and cellular internalization, thus reducing the cytotoxicity of MOFs. The lipid functionalization could inhibit the direct interaction with cells, but lipid layer then enhances the cellular uptake. The internalized MOFs may degrade in the intracellular milieus, which leads to the release of ions and organic linkers as well as cytotoxicity.

### 4.4 Dose

The administered dose of MOFs determines its cytotoxicity as the classical principle in toxicology: dose makes the poison. The toxicity assessment of MOFs is essential in their applications, especially in biomedical science. Therefore, many researchers preferentially investigated the effect of dose on the cytotoxicity of MOFs to make basis for biomedical studies (**Table** **1**).

**Table 1:** Recent studies on the effect of dose on the cytotoxicity of MOFs

MOF	Metal node	Organic linker	Dose [μg mL^−1^]	Cell line	References
ZIF‐8	Zn	2‐Methylimidazole	5–100	HEK293, MDA231, HACAT, RAW264, NIH3T3, MG63	[90]
			10–100	RAW264.7	[91]
			1–750	BEAS‐2B	[100]
GDMU‐4	Zn	[1,1′:3′,1′′‐terphenyl]‐3,3′′,5,5′′‐tetracarboxylic acid	1.25–20	HEK293, Hela	[92]
IRMOF‐3	Zn	2‐aminobenzene‐1,4‐dicarboxylic acid	25–400	PC‐12	[93]
ZIF‐67	Co	2‐Methylimidazole	1–100	BV‐2	[101]
MOF‐74	Cu	2‐hydroxyterephthalic acid	0.01–10	M. aeruginosa	[94a]
			0.01–10	M. aeruginosa	[94b]
HKUST‐1	Cu	1,3,6‐benzenetricarboxylic acid	1–120	*E. coli*, *S. aureus*	[44]
			1–100	HEK293	[102]
			20–60	A. vinelandii	[103]
UiO‐66	Zr	Terephthalic acid	0.1–1000	H9C2	[95]
PCN‐224	Zr	meso‐Tetra(4‐carboxyphenyl)porphine	0.625–20	RAW264.7	[96]
MIL‐100	Fe	1,3,5‐Benzenetricarboxylic acid	10–160	HepG2, HL7702	[97]
MIL‐101	Fe	2‐Aminoterephthalic acid	5–100	NIH/3T3	[98]

ZIFs received great attention in the assessment of cytotoxicity as they were explored as promising drug delivery platform. Recent studies confirmed that ZIF‐8 had high cytotoxicity to cancer cells. Hoop's group investigated the effect of ZIF‐8 on the viability of six kinds of cells and found the values of IC_50_ were around 30 μg mL^−1^.^[^ ^90^ ^]^ ZIF‐8 generated significant ROS and changed cell cycle at no less than 10 μg mL^−1^. Chen's group also found that ZIF‐8 made great toxicity on RAW264.7 cells at ≈22 μg mL^−1^ without the increase of immune factors.^[^ ^91^ ^]^ The cytotoxicity of Zn‐based GDMU‐4 was also evaluated and the safe concentration was below 20 μg mL^−1^.^[^ ^92^ ^]^ Ren's group found that Zn‐based IRMOF‐3 increased cell death from 100 μg mL^−1^, but IRMOF‐3 inhibits macrophage activation at 25 μg mL^−1^.^[^ ^93^ ^]^ These results indicate that the threshold concentration for the cytotoxicity of Zn‐based MOFs is about 30 μg mL^−1^.

Cu‐based MOFs were reported to have great ability in antibacteria and their toxic effects on bacteria were investigated. Fan's group used Cu‐based MOF‐74 as the antibacterial agent to inhibit algae. They confirmed that MOF‐74 induced cytotoxicity at no less than 1 μg mL^−1^ and reached the maximal inhibition effect at 5 μg mL^−1^.^[^ ^94^ ^]^ The toxic effects of Cu‐based HKUST‐1 on *E. coli* and *S. aureus* were studied by Yang's group.^[^ ^44^ ^]^ They found HKUST‐1 was nontoxic to bacteria at low concentrations, and the bacterium death was initiated at high concentrations (900 μg mL^−1^ for *E. coli*; 1200 μg mL^−1^ for *S. aureus*). Meanwhile, HKUST‐1 gave inhibition of enzyme activity for nitrogen‐fixing bacterium *A. vinelandii* from 40 μg mL^−1^ and completely induced growth inhibition at 60 μg mL^−1^.^[94b]^

Zr‐ and Fe‐based MOFs have great chemical stability, permanent porosity, and high surface areas, making them promising materials as drug delivery systems. The cytotoxicity of Zr‐based UiO‐66 was negligible in the concentration range of 0–100 μg mL^−1^.^[^ ^95^ ^]^ However, PCN‐224 gave remarkable toxic effects on RAW264.7 cells at 20 μg mL^−1^, accompanied with ROS generation and membrane rupture.^[^ ^96^ ^]^ The cytotoxicity of Fe‐based MIL‐100 was reported by Ni's group who found the safe concentration of 80 μg mL^−1^ with high cell viability (viability of HL‐7702 > 85%, viability of HepG2 > 91%).^[^ ^97^ ^]^ As for Fe‐based MIL‐101, it had no effect on cell viability for NIH/3T3 cells from 5 to 100 μg mL^−1^, but the autophagy flux was inhibited from 25 μg mL^−1^, indicating that autophagy is a more sensitive index for the cytotoxicity of MOFs.^[^ ^98^ ^]^

## 5 Conclusion and Perspective

Over the past decades, MOFs have become promising candidates in various applications. However, the exposure of MOFs to humans and organisms leads to great health risks. This review has discussed the possible exposure routes of MOFs to humans and the cytotoxicity of MOFs. Based on the existing studies, the key physicochemical factors affecting cytotoxicity of MOFs, that is, chemical composition, size, shape, surface functionalization, and dose, have been identified. It is important to note that cytotoxicity depends on not only a single factor, but also the interplay of multiple factors.

Despite the great progress in the cytotoxicity assessment for MOFs, the studies on long‐term toxicity, the molecular mechanism of cytotoxicity, and the prediction of cytotoxicity are still lacking. Therefore, substantial efforts should be made in the following directions: 1) long‐term cytotoxicity assessment with 3D cell organoid to mimic the internal environment and to monitor the fate of MOFs (such as dissolution and transformation); 2) elucidating the molecular mechanism of cytotoxicity, that is, the molecular initiation event, response pathway, and endpoint of toxicity, with the help of molecular biological methods; 3) predicting the cytotoxicity of MOFs with artificial intelligence to accelerate the experiment and to make it easier to handle the large versatility of MOF structures and compositions;^[^ ^99^ ^]^ and 4) combining in vitro study and in vivo research together to fully understand the biocompatibility of MOFs. Collectively, such efforts are needed to promote the study of the cytotoxicity of MOFs, making it a safer material for the environment and humans.

## Conflict of Interest

The authors declare no conflict of interest.
